# Description of an intramonocytic haemoparasite, *Hepatozoon lainsoni* sp. nov. (Apicomplexa: Adeleorina: Hepatozoidae), infecting *Ameiva ameiva* lizard (Reptilia: Squamata: Teiidae) in northern Brazil

**DOI:** 10.1017/S0031182024000180

**Published:** 2024-04

**Authors:** Rafaela A. P. B. Morais, Ana Paula D. Rodrigues, José Antonio P. Diniz, Letícia Pereira Úngari, Lucia Helena O'Dwyer, Wanderley de Souza, Renato A. DaMatta, Edilene O. Silva

**Affiliations:** 1 Instituto Evandro Chagas, Secretaria de Vigilância em Saúde, Ministério da Saúde, Ananindeua, Pará, Brazil; 2 Instituto de Biociências, Universidade Estadual Paulista, Setor de Parasitologia, Botucatu, São Paulo, Brazil; 3 Laboratório de Ultraestrutura Celular Hertha Meyer, Instituto de Biofísica Carlos Chagas Filho, Universidade Federal do Rio de Janeiro, Rio de Janeiro, Rio de Janeiro, Brazil; 4 Instituto Nacional de Ciência e Tecnologia em Biologia Estrutural e Bioimagem, Universidade Federal do Rio de Janeiro, Rio de Janeiro, Rio de Janeiro, Brazil; 5 Laboratório de Biologia Celular e Tecidual, Centro de Biociências e Biotecnologia, Universidade Estadual do Norte Fluminense Darcy Ribeiro, Campos dos Goytacazes, Rio de Janeiro, Brazil; 6 Laboratório de Biologia Estrutural, Instituto de Ciências Biológicas, Universidade Federal do Pará, Belém, Pará, Brazil

**Keywords:** 18S rDNA, haemoparasite, *Hepatozoon*, lizard, monocyte, tissue stage

## Abstract

Haemogregarine (Apicomplexa: Adeleorina) parasites are considered to be the most common and widespread haemoparasites in reptiles. The genus *Hepatozoon* (Apicomplexa: Adeleorina: Hepatozoidae) can be found parasitizing a broad range of species and, in reptiles, they infect mainly peripheral blood erythrocytes. The present study detected and characterized a haemogregarine isolated from the lizard species, *Ameiva ameiva*, collected from the municipality of Capanema, Pará state, north Brazil. Blood smears and imprints from lungs, brain, heart, kidney, liver, bone marrow and spleen were observed using light microscopy and the parasite was genetically identified by molecular analysis. Morphological, morphometric and molecular data were obtained. Parasite gamonts were found in 49.5% (55/111) of the blood smears from *A. ameiva*, and were characterized as oval, averaging 12.0 ± 0.8 × 5.9 ± 0.6 μm^2^ in size, which displaced the nuclei of parasitized monocytes laterally. Parasite forms resembling immature gamonts were observed in the spleen and bone marrow of the lizards. Furthermore, phylogenetic analyses of 18S rRNA sequences did not reveal gene similarity with other *Hepatozoon* spp. sequences from reptiles. Thus, morphological and molecular analyses have identified a new species of *Hepatozoon* parasite, *Hepatozoon lainsoni* sp. nov., which infects monocytes of the *A. ameiva* lizard.

## Graphical abstract

**Figure:**
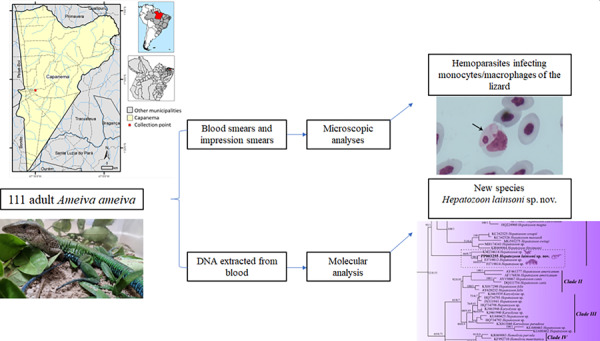

## Introduction

The Phylum Apicomplexa contains a large number of species of protozoan parasites, some of which are recognized as important pathogens that cause human diseases (e.g. *Plasmodium* spp. and *Toxoplasma gondii*) or as animal parasites of considerable economic importance (e.g. *Eimeria* spp. and *Neospora* spp.) (Kemp *et al*., 2013). Apicomplexan parasites have an obligate intracellular existence, and many protozoan parasites also infect the blood cells and organs cells of their vertebrate hosts (Kemp *et al*., 2013; Al-Quraishy *et al*., 2021). The group of haemogregarines (Apicomplexa: Adeleorina) is considered a common parasite in reptiles, where *Hepatozoon* is the most commonly reported parasite. Of note, the gamonts of *Hepatozoon* display an important life-cycle characteristic as they infect peripheral blood erythrocytes of lizards (Telford, 2009).

*Hepatozoon* species are heteroxenous parasites, implying a complex variety of life cycles involving vertebrate and invertebrate hosts (ticks, mosquitoes, flies). Merogony occurs in the vertebrate host, producing gamonts (without sexual dimorphism) that are found within blood cells. Vertebrate infection is believed to occur through ingestion of infected invertebrate hosts and, in reptiles, transmission usually occurs through the ingestion of an infected vector or following predation of a vertebrate (paratenic host) (Smith, 1996; Paperna and Lainson, 2004; Al-Quraishy *et al*., 2021).

Given that parasites may infect a wide range of host species, many haemogregarines have been described in Brazilian reptiles, including caiman (*Hepatozoon caimani* Carini, 1909) and snakes (*Hepatozoon cevapii* O'Dwyer *et al*., 2013, *Hepatozoon massardii* O'Dwyer *et al*., 2013, *Hepatozoon cuestensis* O'Dwyer *et al*., 2013, *Hepatozoon musa* Borges-Nojosa *et al*., 2017) (Carini, 1909; O'Dwyer *et al*., 2013; Borges-Nojosa *et al*., 2017). In reptiles of the Amazon, *Hepatozoon* infection has been reported in *Boa constrictor* (*Hepatozoon cf. terzii* Paperna and Lainson, 2004), *Caiman caimani* (*Hepatozoon caimani* Carini, 1909) and *Ameiva ameiva* (*Hepatozoon ameivae* Carini and Rudolph, 1912) (Lainson *et al*., 2003*b*; Paperna and Lainson, 2004; Picelli *et al*., 2020).

Although the evaluation of morphological values of the host and parasite life-cycle developmental stages is crucial for the correct identification of parasites, molecular data are also essential for differentiating species and genera (O'Donoghue, 2017). Therefore, using morphological and molecular analyses, the present study reports on the presence of a new species of *Hepatozoon*, *Hepatozoon lainsoni* sp. nov., with the unique characteristic of parasitizing monocyte/macrophage cells in the *A. ameiva* lizard.

## Materials and methods

### Lizards

A total of 111 adult *A. ameiva* (Lepidosauria, Teiidae) were hand captured at the municipality of Capanema, Pará state, northern Brazil (01°11′37.6″S, 047°10′01.4″). Lizards were maintained as described by Silva *et al*. (2004).

### Morphological and morphometric analyses

The lizards were restrained manually, and their blood was collected by cardiac puncture into heparinized (100 U mL^−1^) 1 mL syringes. The blood smears and the impression smears (imprints) of the lungs, brain, heart, kidney, liver, bone marrow and spleen were air-dried, fixed with absolute methanol and stained with 10% Giemsa Methylene Blue Eosin Merck^®^ diluted in distilled water (pH 7.0) for 50 min, according to Eisen and Schall (2000), to allow analysis of the different parasite forms and stages. The samples were examined, and the infected cells were photographed with a Zeiss Axiophot microscope using a 100× immersion objective. Prevalence was estimated as the proportion of infected hosts, expressed as a percentage. The intensity of parasitaemia in blood monocytes was graded according to Silva *et al*. (2004) as negative, low-level infected, medium-level infected and highly infected.

Measurements of the length, width and area of the gamonts and host cells (infected and uninfected) were performed. Morphometric data are presented in micrometres (μm), and for each metric, the averages, ranges and standard deviations were also calculated. Measurements of cells were carried out using a 100× oil immersion objective on a Zeiss Axiophot microscope, calibrated with a stage micrometre. Unstained samples were analysed by differential interference contrast (DIC) microscopy.

### Molecular analysis

DNA was extracted from blood using an Illustra blood genomicPrep Mini Spin Kit (GE Healthcare, São Paulo, Brazil) following the manufacturer's instructions. The 18S rDNA gene was amplified with 2 pairs of primers, HepF300/Hep900 (600 bp) primers (Ujvari *et al*., 2004) and 4558/2733 (1000 bp) primers (Mathew *et al*., 2000) to detect the *Hepatozoon* species; reactions were carried out under conditions established by O'Dwyer *et al*. (2013) and Netherlands *et al*. (2014). To check for possible contamination, nuclease-free water was used as a negative control. A blood sample from a *Crotalus durissus terrificus* snake that previously tested positive for *Hepatozoon* spp. was used as the positive control (supplied by the Laboratory of Parasitology of the Institute of Biosciences, UNESP-IB). All reactions were carried out in a Mastercycler pro (Eppendorf, São Paulo, Brazil) thermocycler with the following programme: 94°C for 3 min, 35 cycles of 94°C for 45 s, 50°C for 60 s, and 72°C for 60 s, followed by a final 7 min extension at 72°C.

The amplification products were subjected to electrophoresis at 80 V in a 1.5% agarose gel, stained with Gel Red, and observed using an ultraviolet transilluminator. The products of interest were purified by adding 2 μL of ExoSAP IT enzyme (GE Healthcare) to 5 μL of each polymerase chain reaction product, according to the manufacturer's recommendations. Amplicons were then sequenced using a 3500 Genetic Analyzer capillary sequencer (Applied Biosystems; Foster City, California, U.S.A) and a BigDye Terminator Cycle Sequencing Ready Reaction Kit v.3.1 (Applied Biosystems; Foster City, California, U.S.A), according to the manufacturer's recommendations. A consensus sequence alignment was performed from the forward and reverse electropherograms using BioEdit software version 7.0.9 (Hall, 1999). The sequences obtained (589 and 1052 nt) from *A. ameiva* (GenBank numbers PP003255 and PP003256) were compared among them and with those of other *Hepatozoon* isolates available in the GenBank.

Sequences were aligned with the MUSCLE algorithm using Geneious v.7.1.3 (Kearse *et al*., 2012) for Bayesian inference (BI) and maximum-likelihood (ML) analyses. From this alignment, to compare the 18S rRNA gene in *Hepatozoon* isolates, a pairwise distance (*p*-distance) matrix was used (Table 1). For ML phylogeny, JModelTest v.2.1.10 (Darriba *et al*., 2012) was used to identify the best evolutionary model. Based on the Akaike information criterion, the TVM + G model was chosen for ML analyses. The phylogeny of the parasite was inferred using PhyML v.3.0 (Guindon *et al*., 2010) with 1000 replicate bootstraps (>50%). BI was carried out using MrBayes implemented from the computational resource CIPRES (Miller *et al*., 2010), and analysis was run with the nucleotide substitution model GTR + I + G. To search with the Markov chain Monte Carlo method, chains were run with 10 000 000 generations, saving 1 tree every 1000 generations. On the burn-in, the first 25% of generations were discarded, and the consensus trees were estimated using the remaining trees. Bayesian posterior probability cutoff was considered to be >50%. The trees (BI and ML) were visualized and edited using the FigTree v1.3.1 software program (Rambaut, 2012). The sequences from the phylogenetic tree, their hosts and their GenBank accession numbers are shown in Table 2. The pairwise distance (*p*-distance) and gene similarity were executed by the MEGA 7 program (Kumar *et al*., 2016). A matrix was used to compare the interspecific divergence between the species of *Hepatozoon* sequences isolated from lizards.

**Table 1.** Shaded matrix (upper) shows the similarity percentage (%) of the nucleotide sequences and the unshaded matrix (lower) shows the pairwise distance (*p*-distance) among *Hepatozoon* spp. isolates from lizards

Isolate	1	2	3	4	5	6	7	8	9	10	11	12	13	14	15	16	17	18	19	20
1. This study		96.74	96.72	97.19	97.66	96.72	96.02	96.25	97.89	97.89	91.47	92.51	96.60	96.49	97.89	96.25	95.55	96.02	96.96	95.32
2. MN833640	0.033																			
3. KM234612	0.031																			
4. KM234613	0.026																			
5. KM234616	0.021																			
6. KM234618	0.031																			
7. KM234650	0.038																			
8. KU680423	0.029																			
9. KU680459	0.019																			
10. KU680460	0.019																			
11. KU680462	0.075																			
12. KU680463	0.067																			
13. KU680466	0.031																			
14. KU680465	0.031																			
15. HQ734806	0.019																			
16. HQ734792	0.029																			
17. HQ734793	0.036																			
18. HQ734798	0.031																			
19. AY252108	0.028																			
20. JX531941	0.039																			

Sequence length: 1052 nt.

**Table 2.** Hosts, localities and GenBank accession numbers for the 18S rDNA sequences of *Hepatozoon* spp., *Haemogregarina* spp. and *Hemolivia* spp. used in the phylogenetic analyses (except for the sequence from this study and outgroup)

Parasite	Host	Locality	GenBank no.
*Haemogregarina pellegrini*	*Platysternon megacephalum*	China	KM887509
*Haemogregarina sacaliae*	*Sacalia quadriocellata*	Vietnam	KM887507
*Haemogregarina stepanowi*	*Emys orbicularis*	Bulgaria	KF257928
*Haemogregarina balli*	*Chelydra serpentina serpentina*	Canada	HQ224959
*Haemogregarina podocnemis*	*Podocnemis unifilis*	Brazil	MF476204
*Hemolivia parvula*	*Kinixys zombensis*	South Africa	KR069083
*Hemolivia mauritanica*	*Testudo marginata*	Greece	KF992710
*Hemolivia* sp.	*Rhinoclemmys pulcherrima manni*	Nicaragua	KF992714
*Hepatozoon ewingi*	*Haemaphysalis bancrofti*	Australia	MG593275
*Hepatozoon* sp.	*Podarcis vaucheri*	Morocco	HQ734793
*Hepatozoon* sp.	*Algyroides marchi*	Spain	JX531941
*Hepatozoon* sp.	*Atlantolacerta andreanskyi*	Morocco	HQ734798
*Hepatozoon* sp.	*Tarentola angustimentalis*	Spain	KU680423
*Hepatozoon* sp.	*P. vaucheri*	Morocco	HQ734792
*Hepatozoon* sp.	*Tarentola mauritanica*	Morocco	KU680463
*Hepatozoon* sp.	*T. mauritanica*	Morocco	KU680462
*Hepatozoon felis*	*Panthera leo*	India	KX017290
*H. felis*	*Felis catus*	Spain	AY620232
*Hepatozoon americanum*	*Amblyomma maculatum*	United States	AF176836
*H. americanum*	*Dusicyon thous*	Brazil	AY461377
*Hepatozoon canis*	*Canis lupus familiaris*	Sudan	DQ111754
*H. canis*	*Vulpes* sp.	Spain	AY150067
*Hepatozoon* sp.	*Dromiciops gliroides*	Chile	FJ719814
*Hepatozoon* sp.	*D. gliroides*	Chile	FJ719813
*Hepatozoon* sp.	*Amblyomma fimbriatum* from *Varanus panoptes*	Australia	EU430234
*Hepatozoon* sp.	*Aponomma varanense* from *Ophiophagus hannah*	Thailand	JQ670908
*Hepatozoon* sp.	*Hemidactylus mabouia*	Brazil	KM234616
*Hepatozoon ayorgbor*	*Python regius*	Ghana	EF157822
*Hepatozoon* sp.	*Tarentola deserti*	Algeria	KU680459
*Hepatozoon* sp.	*T. deserti*	Morocco	KU680460
*Hepatozoon* sp.	*T. mauritanica*	Morocco	HQ734806
*Hepatozoon* sp.	*T. mauritanica*	Morocco	KU680466
*Hepatozoon* sp.	*T. mauritanica*	Morocco	KU680465
*Hepatozoon* sp.	*Akodon* sp.	Brazil	KU667308
*Hepatozoon* sp.	*Oplurus* sp.	Madagascar	KM234650
*Hepatozoon caimani*	*Caiman crocodilus yacare*	Brazil	MF322539
*H. caimani*	*C. crocodilus yacare*	Brazil	MF322538
*Hepatozoon* sp.	*Boiga* sp.	Australia	AF297085
*Hepatozoon* sp.	*Ornithodoros atacamensis*	Chile	MH174343
*Hepatozoon* sp.	*Phyllopezus periosus*	Brazil	KM234614
*Hepatozoon massardii*	*Crotalus durissus terrificus*	Brazil	KC342526
*Hepatozoon cevapii*	*C. durissus terrificus*	Brazil	KC342525
*Hepatozoon fitzsimonsi*	*K. zombensis*	South Africa	KR069084
*Hepatozoon* sp.	*Phyllopezus pollicaris*	Brazil	KM234613
*Hepatozoon* sp.	*H. mabouia*	Brazil	KM234618
*Hepatozoon musa*	*Philodryas nattereri*	Brazil	KX880079
*H. musa*	*Epicrates crassus*	Brazil	MF497767
*Hepatozoon cuestensis*	*C. durissus terrificus*	Brazil	KC342524
*H. cuestensis*	*C. durissus*	Brazil	MF497769
*Hepatozoon* sp.	*Boa constrictor*	Brazil	MF497768
*Hepatozoon* sp.	*P. pollicaris*	Brazil	KM234612
*Hepatozoon* sp.	*Varanus scalaris*	Australia	AY252108
*Hepatozoon cecilhoarei*	*Philothamnus natalensis natalensis*	South Africa	MG519504
*Hepatozoon angeladaviesae*	*Philothamnus hoplogaster*	South Africa	MG519501
*Hepatozoon sipedon*	*Nerodia sipedon sipedon*	Canada	JN181157
*Hepatozoon* sp.	*Rana esculenta*	France	HQ224960
*Hepatozoon catesbianae*	Bullfrog	Canada	AF176837
*Hepatozoon ixoxo*	*Sclerophrys* sp.	South Africa	KP119772
*Hepatozoon theileri*	*Amietia quecketti*	South Africa	KP119773

## Results

**Figure 1.**
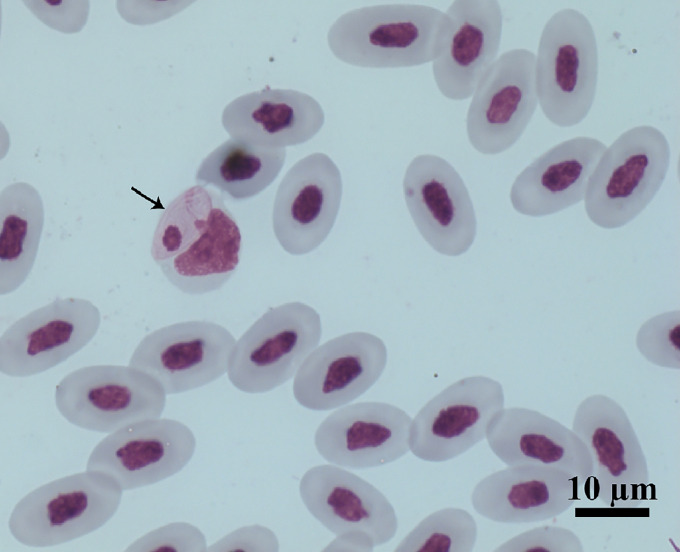
Intramonocytic gamonts (arrows) of *Hepatozoon lainsoni* sp. nov. from peripheral blood of the lizard *Ameiva ameiva* stained with Giemsa. Scale bar: 10 μm.

**Figure 2.**
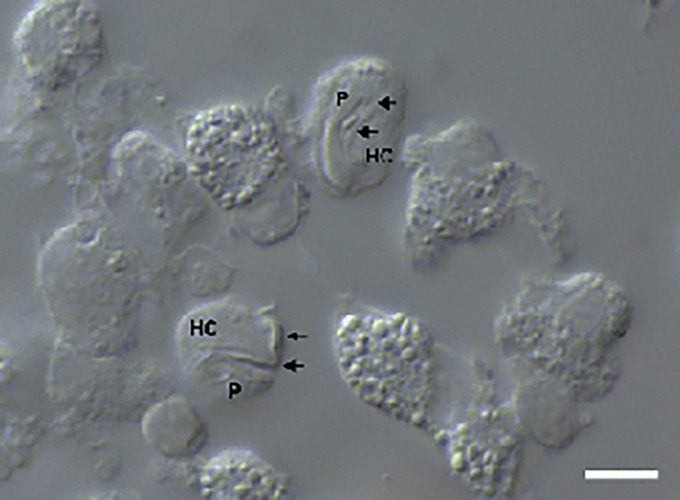
Intramonocytic gamonts (arrows) of *H. lainsoni* sp. nov. from peripheral blood of the lizard *A. ameiva* observed by DIC microscopy. HC, host cell; P, parasite. Scale bar: 10 μm.

**Figure 3.**
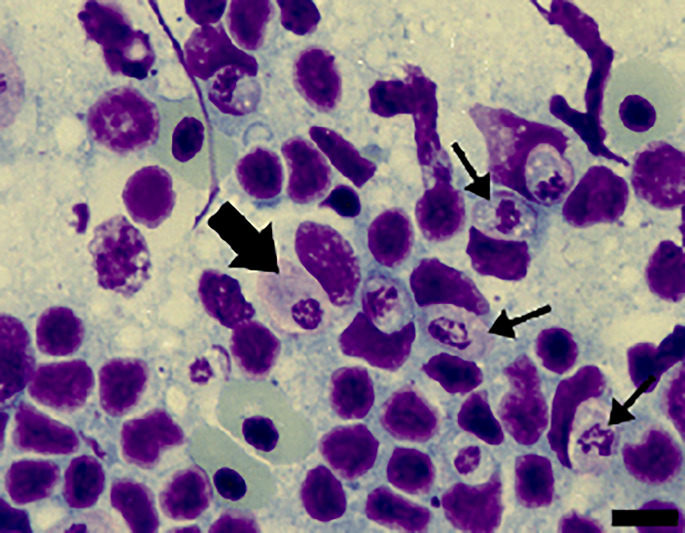
Cells from the spleen imprints stained with Giemsa from *A. ameiva* lizards infected with the parasite. Immature gamonts (arrows) and mature gamonts (big arrow). Scale bar: 10 μm.

Of the 111 *A. ameiva* lizards screened, 55 (49.5%) showed positivity for haemoparasites infecting monocytes in the peripheral blood (Figs 1 and 2) and organ imprints (Fig. 3). The level of infection was usually low (up to 2 parasites per microscopic field), although 5 (9.1%) of the lizard population presented high-level parasitaemia (above 3 parasites per microscopic field). Developmental stages were observed in the spleen (Fig. 3) and bone marrow (not shown), but not detected in other organs. Through morphological, morphometric and molecular evaluation, a previously undescribed species of haemogregarine parasite was identified; this species belongs to the genus *Hepatozoon* Miller, 1908 (Adeleorina: Hepatozoidae).

### Species description

#### Phylum Apicomplexa Levine, 1970 Class Conoidasida Levine, 1988 Subclass Coccidia Leuckart, 1879 Order Eucoccidiorida Léger, 1911 Suborder Adeleorina Léger, 1911 Family Hepatozoidae Wenyon, 1926 Genus *Hepatozoon* Miller, 1908 *Hepatozoon lainsoni* sp. nov. Morais and Silva, 2023

### Taxonomic summary

*Type-host: Ameiva ameiva* (Linnaeus, 1758) (Squamata: Teiidae: Teiinae).

*Type locality:* In areas of the Capanema municipality, Pará state, northern Brazil (01°11′37.6″S, 047°10′01.4″W).

*Site of infection:* Monocytes and macrophages from peripheral blood, spleen and bone marrow, respectively.

*Vector:* Remains unknown; we speculate that it is an external blood-sucking vector, such as a tick species.

*Etymology*: The species is named after the parasitologist, Ralph Lainson, who extensively researched the protozoan parasites of the Brazilian Amazon, and who described this parasite in a preliminary note (Lainson *et al*., 2003*a*).

*Prevalence:* Of the 111 lizards analysed, 55 (49.5%) were positive.

*Material deposited:* Hapantotypes, 1 blood smear, 1 imprint of the spleen and 1 imprint of bone marrow from the *A. ameiva* lizard were deposited in the collection of the National Institute of Amazonian Research (INPA), Manaus, Brazil [INPA 24].

*Gene sequence:* The 18S rRNA gene sequences (589 and 1052 bp), obtained from the blood of *A. ameiva*, were deposited in the GenBank, under accession numbers PP003255 and PP003256.

### Morphological and morphometrical analysis

*Gamonts*: Gamonts were observed exclusively in monocytes of infected lizards. No division stages were detected in blood films. Gamonts were oval and conspicuous in the monocyte cytoplasm, averaging 10.0–13.5 × 4.5–7.7 (12.0 ± 0.8 × 5.9 ± 0.6) μm^2^ (*n* = 50) in size (Table 3), and with an area of 36.9–74.1 (57.9 ± 8.2) μm^2^ (*n* = 50). The parasite cytoplasm was stained greyish-blue or pale pink with thin visible reddish staining at the membrane. Some gamonts contained numerous magenta-staining granules. Nuclei were small, averaging 1.9–5.3 × 1.5–4.0 (3.6 ± 0.8 × 2.6 ± 0.5) μm^2^ (*n* = 50) in size, with an area of 3.2–11. (6.9 ± 1.7) μm^2^ (*n* = 50); they were rounded or irregular shaped, demonstrated dark purple staining and were located at 1 end of the parasite (Fig. 1). DIC microscopy revealed a thin body, with a prolongation, that was doubled on itself and with a nucleus situated in the middle of the parasite (Fig. 2).

**Table 3.** Measurements (μm) of gamonts of *Hepatozoon lainsoni* sp. nov. and *Hepatozoon* sp., infecting lizards from Brazil

*Hepatozoon* species	Vertebrate host	Length (μm)	Width (μm)	Host cell type	References
*Hepatozoon tupinambis*	*Tupinambis teguixim*	16	6	Erythrocyte	Laveran and Salimbeni (1909)
*Hepatozoon missoni*	*T. teguixim*	9	3	Erythrocyte	Carini (1909)
*Hepatozoon cnemidophorus*	*Cnemidophorus* sp.	17	4	Erythrocyte	Carini (1941)
*Hepatozoon sinimbui*	*Iguana iguana*	NI	NI	Erythrocyte	Carini (1942)
*Hepatozoon ameivae*	*Ameiva* sp.	13	4	Erythrocyte	Carini and Rudolph (1912)
*H. lainsoni*	*Ameiva ameiva*	12	6	Monocyte	Present study

NI, not informed.

*Host cell*s: Host cells were enlarged. Parasitized monocytes measured 11.3–20.9 × 8.9–16.0 (14.9 ± 2.1 × 12.5 ± 7.0) μm^2^ (*n* = 50) in size, with an area of 87.2–225.6 (146.2 ± 30.9) μm^2^ (*n* = 50). Nuclei of parasitized monocytes measured 7.3–14.3 × 2.4–8.9 (10.7 ± 1.6 × 5.6 ± 1.4) μm^2^ (*n* = 50) in size, with an area of 32.5–88.4 (48.7 ± 10.2) μm^2^ (*n* = 50). Gamonts displace the nuclei laterally, occasionally becoming deformed and flattened by the parasite. Numerous granules in the cytoplasm were occasionally seen. Uninfected monocytes measured 9.6–17.2 × 8.3–15.3 (13.8 ± 1.6 × 12.2 ± 1.5) μm^2^ (*n* = 50) in size, with an area of 67.6–186.3(139.3 ± 24.8) μm^2^ (*n* = 50); and nuclei measured 7.8–12.3 × 4.4–10.3 (10.2 ± 1.1 × 7.4 ± 1.4) μm^2^ (*n* = 50) in size, with an area of 31.6–86.4 (61.8 ± 13.2) μm^2^ (*n* = 50).

*Tissue developmental stages (mature and immature gamonts)*: Tissue stages in the spleen and bone marrow were seen. Sometimes macrophages of the spleen presented 2 parasites in a single cell, with very abundant forms seen in the spleens of some infected lizards (Fig. 3). Spleen and bone marrow of some infected lizards showed immature gamonts, characterized by an elliptical shape, which measured 7.4–9.7 × 5.6–6.9 (8.3 ± 0.8 × 6.4 ± 0.4) μm^2^ (*n* = 10), and with an area of 29.0–51.0 (43.6 ± 7.4) μm^2^ (*n* = 10). These gamonts had purplish or pinkish cytoplasm with bright granules and fragmented nuclei that occupied the central position or were displaced towards one of the extremities. Another gamont form presented an oval body measuring 8.4–13.5 × 4.4–6.4 (11.3 ± 1.7 × 5.5 ± 0.7) μm^2^ (*n* = 10) and with an area that measured 35.2–63.6 (47.7 ± 8.4) μm^2^ (*n* = 10); this form had pinkish cytoplasm, with rounded or fragmented nuclei that were displaced towards one of the extremities of the parasite, representing mature gamonts. These stages have variable sizes (Fig. 3).

Morphological and molecular analyses identify a new species of *Hepatozoon* (Apicomplexa: Adeleorina: Hepatozoidae).

### Remarks

Prior to this study, *Hepatozoon* spp. were reported infecting erythrocytes of reptiles, and occasionally leucocytes. The haemogregarine described herein appears to infect monocytes of the peripheral blood and division stages were seen only in internal organs. Gamonts of *H. lainsoni* sp. nov. presented differences in morphological and morphometrical characteristics at the blood and tissues stages, when compared to other species of *Hepatozoon* described in *A. ameiva* of Minas Gerais and the Amazonas states in Brazil (Carini and Rudolph, 1912; Picelli *et al*., 2020), or any other species currently infecting lizard hosts (Table 4).

**Table 4.** Measurements (μm) of host cells and gamonts of *H. lainsoni* sp. nov. observed in infected *A. ameiva* lizards from Pará state, Brazil

	*n*	Area (μm^2^)	Length (μm)	Width (μm)
Parasite	50	36.9–74.1 (±8.2)	10.0–13.5 (±0.8)	4.5–7.7 (±0.6)
Parasite nucleus	50	3.2–10.1 (±1.7)	1.9–5.4 (±0.78)	1.5–4.0 (±0.52)
Infected monocyte	50	87.2–225.6 (±31.0)	11.3–20.9 (±2.1)	8.9–16.0 (±7.0)
Infected monocyte nuclei	50	32.5–88.4 (±10.2)	7.3–14.3 (±1.6)	2.4–8.9 (±1.4)
Uninfected monocyte	50	67.6–186.3 (±24.9)	9.6–17.2 (±1.6)	8.3–15.3 (±1.5)
Uninfected monocyte nuclei	50	31.6–86.4 (±13.2)	7.8–12.3 (±1.1)	4.4–10.3 (±1.4)

±, standard deviation.

Erythrocytic haematozoans of *A. ameiva* were described by Lainson *et al*. (2003*a*) to be slim parasites measuring 13.1 × 3.0 μm^2^ and with a nucleus located at the parasite's curved extremity, leading the authors to compare these with haematozoans of *H. ameivae* from Minas Gerais, Brazil (Carini and Rudolph, 1912). In 2020, Picelli *et al*. redescribed *H. ameivae* in the peripheral blood of the same host in the Central Amazon by morphological, morphometrical and phylogenetic analyses. Gamonts were found infecting erythrocytes. The parasites were described to be measuring 14.2 × 4.5 μm^2^, with curved extremities and with nuclei that were rounded or irregular in shape, and measuring 4.9 × 3.2 μm^2^.

With regards to other species from host lizards, *H. lainsoni* sp. nov. have a similar size range to those of *H. caimani* Carini, 1909 (12.15 × 4.3 μm^2^) from the caiman *Caiman c. crocodilus* (Lainson *et al*., 2003*b*), *Hepatozoon terzii* Sambon and Seligman, 1907 (12.3 × 4.3 μm^2^) from snake *B. constrictor* (Paperna and Lainson, 2004), *Hepatozoon quagliattus* Úngari, Netherlands, Silva and O'Dwyer, 2021 (13.58 × 6.22 μm^2^) from the sleep snake *Dipsas mikanii* (Úngari *et al*., 2021) and are smaller than *H. musa* Borges-Nojosa, Borges-Leite, Maia, Zanchi-Silva, da Rocha Braga and Harris, 2017 (18.9 × 3.8 μm^2^) from snake *Philodryas nattereri* (Borges-Nojosa *et al*., 2017).

The new species, *H. lainsoni* sp. nov., is characterized by elongated gamonts, which appear to have an oval shape and are not bent at the end. Gamonts were surrounded by a delicate capsule that increased the size of the host cell. Additionally, *H. lainsoni* sp. nov. have small nuclei that are located at 1 end of the parasite. *Hepatozoon lainsoni* sp. nov. share certain characteristics with *H. caimani*, since the gamonts doubled by themselves, and had a visible capsule where both ends were rounded. However, *H. caimani* exhibit gamont nuclei that are located laterally in the parasite, and extracellular parasites can be seen occasionally in the slim form.

In contrast to *H. lainsoni* sp. nov., gamonts of *H. musa* are longer and thinner, and are curved at both ends with their nuclei in the central position. Mature gamonts of *H. quagliattus* are elongated with a thin parasitophorous vacuole and are slightly curved at 1 end; the gamont nucleus is larger than that of *H. lainsoni* sp. nov. and slightly displaced to 1 side of the parasite.

With regards to tissue stages, *H. lainsoni* sp. nov. exhibit immature and mature gamonts in the spleen and bone marrow, with high parasitaemia in the spleen macrophages of some lizards. These gamonts presented a rounded body, colourless cytoplasm and were variable in size.

### Molecular analysis

**Figure 4.**
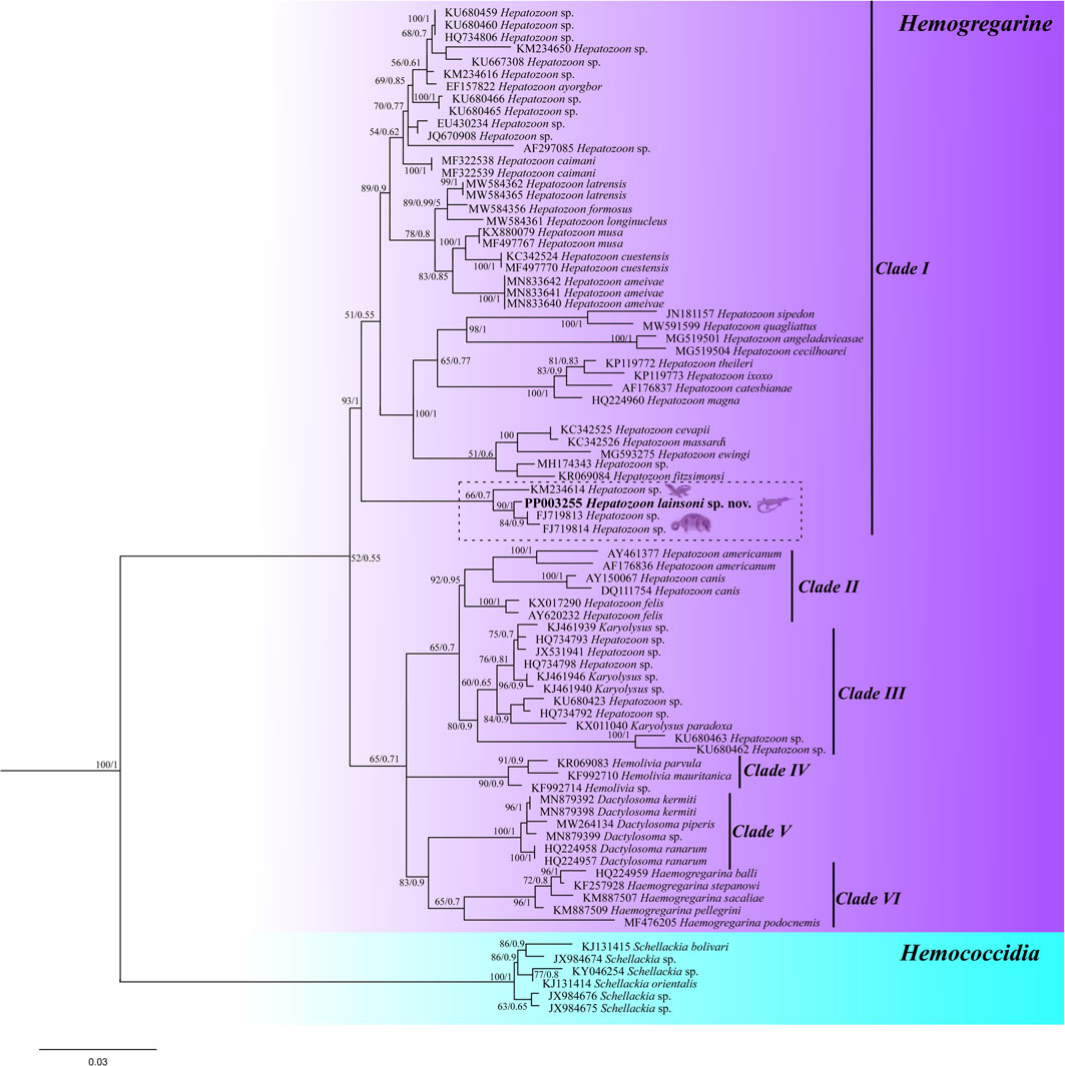
Phylogenetic tree of *Hepatozoon* spp. based on 18S rRNA region gene (1052 bp – sequence length). The topology was identical across ML and BI with supported node value clades.

Both the newly amplified sequences from this study have shown 100% similarity among them. A large sequence (1052 nt) was used to construct BI and ML trees. The trees resulted in identical topologies with supported node value clades (Fig. 4). The trees are formed by haemogregarine isolates, haemogregarine (Haemogregarinidae, Karyolysidae and Hepatozoidae) and haemococcidia isolates as an outgroup. The main clade was subdivided into 6 subclades. The 1st subclade (clade I) comprises *Hepatozoon* isolates from reptiles, anurans and marsupials, including the isolate from this study; furthermore, the isolate from this study grouped together with 3 isolates, 2 from marsupial hosts (FJ719813/FJ719814) and 1 from lizards (KM234614). The 2nd subclade (clade II) comprises *Hepatozoon* isolates from mammals; the 3rd subclade (clade III) describes isolates from *Karyolysus* and *Hepatozoon* species from lizards; the 4th subclade (clade IV) presents *Haemolivia* isolates; the 5th subclade (clade V) comprises *Dactylosoma* isolates from anurans and the 6th (clade VI) comprises *Haemogregarina* isolates from turtles.

## Discussion

Species of *Hepatozoon* have a wide geographic distribution, infecting amphibians, reptiles, birds and mammals. To date, species of *Hepatozoon* have been reported mainly infecting erythrocytes of reptiles. However, other species infecting mammals and birds are found mainly infecting leucocytes (Smith, 1996; Godfrey *et al*., 2011; Ebani and Mancianti, 2022). This study provides molecular analysis and morphological and morphometric descriptions of the blood forms and tissue stages of *H. lainsoni* sp. nov., a species that infects monocytes of the lizard *A. ameiva*.

In the present investigation, blood smears from infected lizards usually showed low parasitaemia. A previous study analysing blood from *A. ameiva* reported only 10% of the lizard population with high parasitaemia and a higher relative increase in the population of blood monocytes, many of which were infected with *H. lainsoni* sp. nov. (Lainson *et al*., 2000a; Bonadiman *et al*., 2010). Similarly, Úngari *et al*. (2018) observed low-level parasitaemia in captive snakes from Brazil with positive blood smears. Other studies of *Hepatozoon* sp. in reptiles reported 0.25% parasitaemia in the sleep snake *D. mikanii* (from Goiás state, Brazil) when infected with *H. quagliattus* Úngari and O'Dwyer, 2021 (Úngari *et al*., 2021) and 0.73% parasitaemia in *A. ameiva* from Central Amazonia infected with *H. ameivae* Carini and Rudolph, 1912 (Picelli *et al*., 2020).

The overall prevalence in this study was 49.5%, which is close to that reported in the preliminary note about this parasite infecting *A. ameiva* (41.7%) (Lainson *et al*., 2003*a*). The prevalence of *Hepatozoon* sp. infection observed in our study is in agreement with another study of *A. ameiva* infected with *H. ameivae* Carini and Rudolph, 1912 from Brazil, which found 55.5% of prevalence in 72 lizards in the Amazonas state (Picelli *et al*., 2020). Furthermore, a higher prevalence of the occurrence of *Hepatozoon* sp. infecting free-ranging caimans (*C. crocodilus yacare*) was reported in a study in the state of Mato Grosso, Brazil (70.8%) (Bouer *et al*., 2017) and in snakes (*Python regius*) in Ghana (78.2%) (Sloboda *et al*., 2007), both using blood smear examination. Lower prevalences of 2–17.6% were observed in lacertid hosts from the Maghreb region of North Africa (Maia *et al*., 2011) and in Asian snakes (22.2%) (Haklová *et al*., 2014). Although different methodologies were used in these studies, Maia *et al*. (2011) suggested that infection with *Hepatozoon* is age-related, considering that the prevalence among snakes seems to be higher than among lizards, and that snakes live much longer than lizards, so that age is related to a higher chance of acquiring the infection, which might persist for a long time (Jakes *et al*., 2003; Javanbakht *et al*., 2015).

To date, species of *Hepatozoon* have been reported as infecting the erythrocytes and leucocytes of reptile, mammal and bird hosts (Godfrey *et al*., 2011; O'Donoghue, 2017; Ebani and Mancianti, 2022). In this study, we further characterized a species of *Hepatozoon* infecting monocytes and macrophages. Morphological and morphometric characteristics and phylogenetic analysis distinguished this species from other *Hepatozoon* species of lizards, such as *H. ameivae* Carini and Rudolph, 1912, *Hepatozoon tupinambis* Laveran and Salibeni, 1909, *Hepatozoon missoni* Carini, 1909, *Hepatozoon cnemidophorus* Carini, 1941 and *Hepatozoon sinimbui* Carini, 1942 (Carini, 1909, 1941, 1942; Laveran, 1909). Through phylogenetic analysis, it was possible to observe that the *Hepatozoon* species from this study was grouped closer to *Hepatozoon* from marsupial hosts than lizard hosts. This fact corroborates with the observed morphological data. *Hepatozoon lainsoni* was found infecting defence cells, a characteristic normally found in mammals; while in reptiles it is common to be found in erythrocytes. Therefore, the morphological and molecular data suggest that *H. lainsoni* may have an ancestor closer to mammals than to reptiles.

The morphometric and morphological analysis showed that the average size and tissue stages of *H. lainsoni* sp. nov. closely resembled those previously described by Lainson *et al*. (2003*a*). Gamonts of *Hepatozoon* from this study revealed differences compared to the other species of *Hepatozoon* described in reptiles. In the literature, the *Hepatozoon* species described in *A. ameiva*, *H. ameivae* Carini and Rudolph, 1912 are recognized to have a slim and elongated form, with both rounded and slightly arched extremities, with nuclei located at its curved extremity, and to infect erythrocytes. In contrast, *H. lainsoni* gamonts had an oval shape with rounded extremities and a small nucleus and infected monocytes. Indeed, DIC microscopy revealed the gamont to have a slim body. These data were supported by ultrastructural analysis of the blood forms, which showed an elongated form, with some embracing the monocyte cytoplasm and, sometimes in intimate contact with the host cell nucleus, suggesting a type of nutrient capture (Silva *et al*., 2004). Again, these results corroborate the description of a new species of *Hepatozoon*.

One noticeable characteristic of the *Hepatozoon* species was the alterations in the monocyte host cell. In the present study, enlargement and deformation of the nucleus, which was flattened by the parasite, were observed. Similar morphological alterations in the host monocyte after parasite infection were previously observed by Lainson *et al*. (2003*a*) and Silva *et al*. (2004) using optical and transmission electron microscopy, respectively. Furthermore, accumulation and association of mitochondria and Golgi complex vesicles at the intracellular parasite were also observed by Silva *et al*. (2004). Shape alteration, lateral displacement of the nucleus and enlargement of infected blood cells have already been reported in snakes *Hydrodynastes gigas* and *C. terrificus* infected by *Hepatozoon* sp. in Brazil (Moço *et al*., 2002). In the present study, tissue stages were observed in the spleen and bone marrow, with the presence of immature and mature gamonts in some lizards; merogony and cystic forms were not seen. Indeed, we did not examine the skeletal muscle and intestines of infected lizards, where the occurrence of merogony and tissue cysts containing cystozoites may be possible, as seen in the host cells of mammal hosts infected with *Hepatozoon felis*, *Hepatozoon americanum* and *Hepatozoon silvestris* (Hodžić *et al*., 2017).

Dividing meronts were observed in the lungs and/or liver tissues of reptiles infected with *H. terzii* (Paperna and Lainson, 2004), *Hepatozoon kisrae* (Paperna *et al*., 2002) and *H. quagliattus* (Úngari *et al*., 2021). Conversely, Lainson *et al*. (2003*b*) described *H. caimani* Carini, 1909 in *Caiman c. crocodilus* and detected meronts only in the lamina propria of experimentally infected caimans, suggesting that merogony is limited to the lamina propria of the small intestine and justifying the failure of other authors to detect meronts in the viscera of naturally infected caimans. It is probable that a similar pattern of merogonic stages may occur in *H. lainsoni* and further investigative endeavours are needed to clarify this.

Therefore, these discoveries underscore the need for expanded research on haemoparasite infections in lizards and other reptiles, especially in the Brazilian Amazon, which represents a geographic region with a great diversity of hosts and the possibility of finding genetically distinct haemogregarines.

## Data Availability

Representative sequences related to this article have been deposited in the GenBank under accession numbers PP003255 and PP003256.
